# Influence of the Microstructure and Silver Content on Degradation, Cytocompatibility, and Antibacterial Properties of Magnesium-Silver Alloys In Vitro

**DOI:** 10.1155/2017/8091265

**Published:** 2017-06-22

**Authors:** Zhidan Liu, Ronald Schade, Bérengère Luthringer, Norbert Hort, Holger Rothe, Sören Müller, Klaus Liefeith, Regine Willumeit-Römer, Frank Feyerabend

**Affiliations:** ^1^Institute for Material Research, Helmholtz-Zentrum Geesthacht, Max-Planck-Str. 1, 21502 Geesthacht, Germany; ^2^Institute for Bioprocessing and Analytical Measurement Techniques e.V. (iba), Rosenhof, 37308 Heilbad Heiligenstadt, Germany; ^3^Extrusion Research and Development Center, Chair Metallic Materials, TU Berlin, Gustav-Meyer-Allee 25, 13355 Berlin, Germany

## Abstract

Implantation is a frequent procedure in orthopedic surgery, particularly in the aging population. However, it possesses the risk of infection and biofilm formation at the surgical site. This can cause unnecessary suffering to patients and burden on the healthcare system. Pure Mg, as a promising metal for biodegradable orthopedic implants, exhibits some antibacterial effects due to the alkaline pH produced during degradation. However, this antibacterial effect may not be sufficient in a dynamic environment, for example, the human body. The aim of this study was to increase the antibacterial properties under harsh and dynamic conditions by alloying silver metal with pure Mg as much as possible. Meanwhile, the Mg-Ag alloys should not show obvious cytotoxicity to human primary osteoblasts. Therefore, we studied the influence of the microstructure and the silver content on the degradation behavior, cytocompatibility, and antibacterial properties of Mg-Ag alloys in vitro. The results indicated that a higher silver content can increase the degradation rate of Mg-Ag alloys. However, the degradation rate could be reduced by eliminating the precipitates in the Mg-Ag alloys via T4 treatment. By controlling the microstructure and increasing the silver content, Mg-Ag alloys obtained good antibacterial properties in harsh and dynamic conditions but had almost equivalent cytocompatibility to human primary osteoblasts as pure Mg.

## 1. Introduction

The clinical application of biodegradable implant and prosthesis has shown rapid growth to keep with the demands of a rapidly aging population. However, implant-associated orthopedic surgery infections are common postoperative wound infections and can cause biofilm formation on the implants or bones [1, 2]. Biofilms are resistant to antibiotics and can protect bacteria from host immune mechanisms. Once a biofilm has formed, the only treatment is to remove the implant and the diseased tissue [3–5]. Prevention is the preferred method to address the growing problem of implant-associated infections [6, 7].

Pure magnesium (pure Mg) and its alloys, as potential biodegradable implant materials, have the advantage of not requiring removal after bone tissue healing [8]. Therefore, infection caused by a second surgery can be avoided. In vitro, pure Mg exhibited some antibacterial properties due to its alkaline pH [9–11]. In the early stage of degradation, it can create an alkaline environment, which is adverse to the survival and reproduction of bacteria [12, 13]. However, it is not clear whether these changes will occur in vivo, although it was shown that pure Mg induces osteoblasts and suppresses bacteria in a chronically infected rabbit tibial osteomyelitis model [14]. However, the length of time that an effective antibacterial concentration maintained in the local position is not sufficient, which will influence the resistance to infection and will affect osteomyelitis treatment [14, 15]. One cause of these effects is that the degradation rate of pure Mg and magnesium alloys in vivo is lower than that in vitro [16–18]. In this case, a high pH cannot be maintained, so it sounds unrealistic for pure Mg or its alloys to achieve long-term inhibition to bacteria.

Silver (Ag) has effective antibacterial properties and has been used to treat burns and chronic wounds for centuries [19]. Silver nanoparticles (AgNPs) and silver ions can bind to proteins, change the membrane of bacteria, interfere with DNA expression, create reactive oxygen species (ROS), and affect thiol group compounds that exist in respiratory enzymes to inhibit respiratory processes [20–22]. The emergence of antibiotic-resistant strains of bacteria has promoted the use of metallic silver to prevent infections of indwelling devices [20]. There are cases of silver applications that focus on the antibacterial properties, for example, wound dressing, bone cement, and megaprosthesis [23–25]. Silver-coated megaprosthesis can release silver ions and reduce the infection rate compared to the group without silver [25]. In addition, metallic silver can stimulate osteogenic differentiation [26].

However, the accumulation of a high amount of silver in the human body can cause argyria or argyrosis, which results from the deposition of significant amounts of insoluble silver precipitates in the dermis of the skin and the cornea or conjunctiva of the eyes [27, 28]. However, no pathological damage to tissues can be observed. The threshold amount of silver that can evoke argyria ranges from 3.8 to 5 g or even 10 g over the whole lifetime of adults [29]. The total body silver concentration that can cause argyria is 1 g for children under 10 years old [30]. Hence, the application of silver in the human body should consider these limitations. In clinical course, the amount of silver coated on megaprosthesis ranges from 0.4 to 1.69 g in adult patients [25]. However, no relevant evidence shows that such a low amount of silver in the human body or chronic silver exposure can cause pathological changes in any tissue or organ [27–29, 31]. Moreover, the loss of cell viability in vitro due to metallic silver or silver compounds is dose dependent [26, 32, 33]. Metallic silver has a lower risk of toxic effects compared with soluble silver compounds [34].

How to avoid or treat orthopedic implant contamination and biofilm formation is a complicated issue [35]. Novel, biodegradable magnesium alloys with antimicrobial properties are desirable considering the surgical contamination and appearance of multiresistance bacteria. Many methods have been studied, for example, coating and surface morphology, to endow permanent implants or even magnesium alloys the function of suppressing bacteria or reducing bacterial adhesion [20, 36–39]. However, maintaining long-term and stable prevention of implant-associated infection remains a problem [40]. In addition, the current methods cannot maintain the long-term antibacterial properties of biodegradable magnesium alloys.

In this study, metallic silver was alloyed with pure Mg so that the silver could be released continuously and react with bacteria if the bacteria attach to the surface of Mg-Ag alloy or the surrounding tissue during the whole degradation period. We planned to reach the best antibacterial properties of Mg-Ag alloys by alloying silver inside as much as possible and obtain good cytocompatibility comparable to those of pure Mg by adjusting their microstructure via thermal-mechanical processing and heat treatment. It is expected that biofilms cannot form on the Mg-Ag alloy and surrounding tissue, even in a harsh environment with a large amount of bacteria and under flow conditions, for example, the human body. By this method, it is hoped that implant-associated and recurrent infections can be prevented successfully when Mg-Ag alloy is used as a bone implant in the future.

## 2. Material and Methods

### 2.1. Materials Preparation

Magnesium (99.99 wt%, Xinxiang Jiuli Magnesium Co., Ltd., Xinxiang, China) and silver granules (99.99 wt%, ESG Edelmetall-Handel GmbH & Co. KG, Rheinstetten, Germany) were used for the preparation of Mg-6Ag and Mg-8Ag alloys. Pure magnesium was cut into small pieces and placed in a steel crucible with the corresponding amount (6 wt% or 8 wt%) of silver. The metals were melted at 750°C under a protective atmosphere of 98% Ar (argon) and 2% SF_6_ (sulphur hexafluoride) and then stirred for 30 minutes at 200 rpm. After the temperature decreased to 730°C, the melt was transferred to a permanent steel mould (diameter ø = 120 mm) that was coated inside with the mould release agent ALU-STOP LC25 (Büro für angewandte Mineralogie, Dr. Stephan Rudolph, Tönisvorst, Germany). The mould was held for 15 min at 680°C under a protective atmosphere and was then immersed in flowing room temperature water gradually at a speed of 100 cm/min until the melted magnesium alloy solidified. Pure Mg ingots were recast into cylinder shape following the same solidification procedure. The tops and bottoms of the ingots with shrinkage and impurities were removed. The contents of elements in the Mg-Ag alloys were analyzed by X-ray fluorescence (Bruker AXS S4 Explorer, Bruker AXS GmbH, Germany) and with a Spark Analyser (Spectrolab M, Spektro, Germany).

According to the Mg-Ag phase diagram, silver has low solubility of 15 wt% in magnesium at eutectic temperature which is the lowest melting point of a mixture of components [41]. Homogenization treatment and hot extrusion were performed to acquire a homogeneous microstructure and stable mechanical properties. The homogenization treatment was conducted in a resistance furnace (Linn Elektro Therm AK 40. 06, Bad Frankenhausen, Germany) at 430°C for 16 h, followed by quenching in room temperature water. The ingots were then heated up (285°C for Mg-6Ag and 300°C for Mg-8Ag) and processed by hot extrusion (Strangpresszentrum Berlin, Berlin, Germany), for which the extrusion ratio and the advance rate of stamp were 108 and 0.7 mm/s, respectively. The temperature of the container and steel die was 300°C. The extruded rods (ø = 12 mm) were cut into discs (ø = 10 mm and *h* = 1.5 mm) (Henschel KG, Munich, Germany). T4 heat treatment (solution treatment) of the discs was conducted by placing them in a steel box filled with Ar and holding them in a resistance furnace (Vulcan™ A-550, Dentsply Ceramco, USA) at 450°C for 8 h and before quenching. The discs were ground on sandpaper (2500#, mesh) to remove the oxidation layer caused by heat treatment.

### 2.2. Microstructure Analysis

Samples were prepared by grinding on sandpaper from 220# to 2500#, followed by polishing using water-free OPS (oxide polishing suspension) on a rubber plate. The samples were etched in picric solution (100 mL ethanol, 20 mL distilled water, 6-7 mL glacial acetic, acid and 12–15 g picric acid (99%), all chemicals from Sigma-Aldrich Chemie, Taufkirchen, Germany) and were observed by polarized light microscopy (Leica 020-520.008 DM/LM, Wetzlar, Germany) and scanning electron microscopy (SEM, TESCAN vega 3 SBU, Brno, Czech Republic). The precipitates in the extruded Mg-Ag alloy were characterized by Bruker X-ray diffraction (XRD).

### 2.3. Immersion Test

An optimized in vitro test setup was used for the immersion tests [42, 43]. Cell culture medium (CCM), Dulbecco's Modified Eagle's Medium (DMEM), and GlutaMAX +10% FBS (fetal bovine serum, PAA laboratories, Linz, Austria) were used for the immersion test and cell culture. The cell culture conditions were 5.0% CO_2_, 20% O_2_, 37.0°C, and 97.0% rH (relative humidity). Discs were weighed by a Scaltec SBA32 (Scaltec, Goettingen, Germany) and sterilized ultrasonically in 70% ethanol solution for 30 min. After drying, the discs were transferred to 12-well plates, which were filled with 2 mL CCM in each well, and then incubated in the cell culture conditions for 1 week. The old cell culture medium was replaced by a fresh one after 48 and 120 hours. After the immersion test was carried out for 168 hours, the degradation products were removed by chromic acid (180 g/L in a. dest., Sigma-Aldrich Chemie, Taufkirchen, Germany). The discs were dried in a vacuum box and weighed by the precision electronic balance. The mean degradation rate was calculated using the previously described weight loss method [44].

### 2.4. Cytotoxicity Test

Human primary osteoblasts were selected for the cytotoxicity evaluation. The human primary osteoblasts came from patients undergoing total hip arthroplasty with local ethical committee agreement. Pure Mg and Mg-Ag discs were sterilized ultrasonically in 70% ethanol solution for 30 min. Extracts of pure Mg and Mg-Ag for the MTT assay were prepared by immersing samples into CCM (0.2 g/mL) for 3 days in the cell culture conditions. The concentration of Mg, calcium (Ca), and Ag in the extracts was measured via inductively coupled plasma mass spectrometry (ICP-MS; Agilent 7700x ICP-MS, Waldbronn, Germany). The extracts were further characterized by measuring their pH and osmolality at room temperature using an ArgusX pH meter (Sentron, Roden, Netherlands) and a Gonotec 030-D cryoscopic osmometer (Gonotec, Berlin, Germany), respectively. A 50 *μ*L aliquot of CCM containing 2000 human primary osteoblasts was seeded into each well of 96-well plates. The plates were transferred to the incubator and kept in the cell culture conditions for 24 hours to ensure that the human primary osteoblasts attached to the bottom. The diluted pure Mg and Mg-Ag extracts were prepared by adding CCM at ratios of 1 : 5 and 1 : 10. Then, the old CCM was replaced with fresh CCM (control group), pure Mg and Mg-Ag extracts, and diluted extracts (*n* = 6 for each concentration). After culturing for 3 days, 10 *μ*L 3-(4,5-dimethylthiazol-2-yl)-2,5-diphenyl-tetrazolium bromide solution (MTT) (Sigma-Aldrich, Steinheim, Germany) was added to each well and incubated for 4 hours. Then, 100 *μ*L SDS (sodium dodecyl sulphate) lysis buffer (Sigma-Aldrich Co., LLC, Steinheim, Germany) was added into the wells and incubated overnight. Finally, the values were measured using an ELISA multiwell plate reader (Tecan, Maennedorf, Switzerland) and the background value was removed.

In the adhesion test, pure Mg and Mg-Ag discs were placed in 12-well plates after the discs incubated in CCM in the cell culture conditions for 24 hours. A total of 10^5^ human primary osteoblasts were seeded on the surface of each disc. To ensure that the human primary osteoblasts attached to the surface, the seeded samples were kept in the incubator for 30 min. Then, 12-well plates were slowly filled with 3 mL of fresh CCM and transferred into the incubator and cultured for 3, 6, or 9 days. The CCM was changed every 3 days. The pH and osmolality of the replaced medium were measured. Discs were washed gently in sterilized and distilled water and transferred into wells filled with LIVE/DEAD® Viability/Cytotoxicity Kit (Molecular Probes, Eugene, USA) according to the manufacturer's protocol. After incubation for 20 min, the distribution and viability of human primary osteoblasts on pure Mg and Mg-Ag discs were observed via fluorescent microscope (Nikon Eclipse Ti-S, Tokyo, Japan).

### 2.5. Biofilm Test and Evaluation

**Figure 1:**
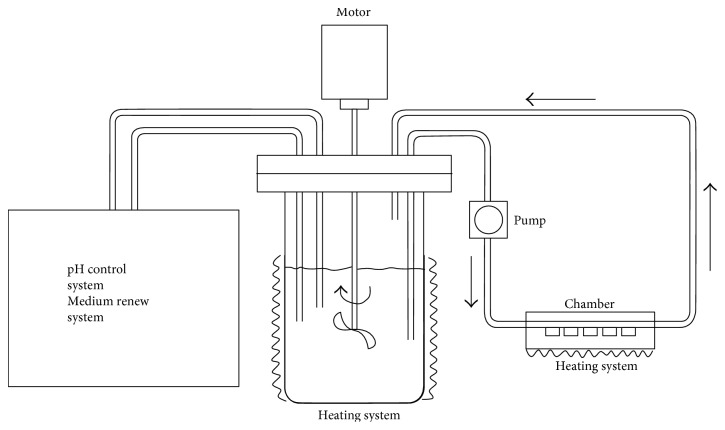
Schematic diagram of the bioreactor system.

The biofilm test was conducted in a bioreactor system (Figure 1). This dynamic system has a cross-flow condition in the chambers, which ensures that the bacteria go through the surface of the discs. The flow rate of the medium in chamber was 0.3 mL/min. These conditions allow the possibility of initial biofilm formation on the discs [45]. During the running time of 15 hours, the temperature and pH were 37°C and 7.2, respectively. All the parameters mentioned above were controlled by the bioreactor system (BioFlo®/CelliGen® 115 (New Brunswick™), Eppendorf AG, New Brunswick, USA). Reference discs (titanium (Ti)) were always used as an internal control for the test. Pure Mg and Mg-Ag alloys were treated with 25.0 kGy gamma sterilization (BBF Sterilisationsservice GmbH, Kernen, Germany) before the biofilm test [44]. The whole test was performed in a microaerophilic and sterilized environment to ensure bacterial activity. The bacteria culture medium (BCM) consisted of nutrient broth (pH = 7.2), 3 g meat extract, 5 g peptone (Sigma-Aldrich Co., LLC, Steinheim, Germany), and 1 L distilled water. Phosphate-buffered saline (PBS, pH = 7.4) was prepared with 8 g NaCl, 0.2 g KCl, 1.47 g Na_2_HPO_4_, 0.24 g KH_2_PO_4_ (Sigma-Aldrich Co. LLC, Steinheim, Germany), and 1 L double-distilled water.

*Staphylococcus aureus* (*S. aureus*, DSM number 20231) and *Staphylococcus epidermidis* (*S. epidermidis*, DSM number 3269) were used in the biofilm test. These bacteria are commonly found in implant-associated orthopedic infections or osteomyelitis [3, 46–48], although there is contention about which is the most common bacteria isolated from clinical infections, especially implant-associated infections [49–51]. The bacteria were provided by the Leibniz Institute DSMZ-German Collection of Microorganisms and Cell Cultures in Germany. The bacteria were cultured overnight and were mixed and imported into the bioreactor system after checking their viability. The density and ratio of the mixed bacteria in medium were 10^6^ /mL and 1 : 1, respectively.

After the bioreactor system ran for 15 hours, all of the discs were removed from the chamber and labelled by adding LIVE/DEAD BacLight™ Bacterial Viability Kit (Thermo Fisher Scientific Inc. (Life Technologies), Eugene, USA). Some discs were observed by confocal laser scanning microscopy (CLSM, LSM 710, Carl Zeiss Microscopy GmbH, Jena, Germany). Images of the whole surface and the local details of the discs were taken by CLSM. The other discs (*n* = 12 for each type of sample) were rinsed gently in distilled water, placed in glass bottles with PBS, and transferred to an ultrasonic bath (SONOREX SUPER 10P, BANDELIN electronic GmbH & Co. KG, Berlin, Germany). A plastic scraper was used to remove the bacteria from the surfaces of the discs under sonication. The PBS solutions containing bacteria were diluted, placed on a counting chamber, and counted using a fluorescence microscope (BX51, Olympus Optical Co. (Europa) GmbH, Hamburg, Germany).

### 2.6. Sample Preparation for SEM and Generation of 3D Images

In adhesion test, the procedures to prepare the SEM samples with human primary osteoblasts were fixation in 2.5% glutaraldehyde solution in buffer (Sigma-Aldrich Co. LLC, Steinheim, Germany) for 2 hours, staining in 1% osmium tetroxide (Sigma-Aldrich Co. LLC, Steinheim, Germany) for 30 min, dehydration for 1 hour using increasing concentrations of 2-propanol (EMSURE®, Darmstadt, Germany) (20%, 40%, 60%, 80%, and 100%), and critical point drying (Leica EM CPD030, Bal-TEC AG, Balzers, Liechtenstein). Then, the samples were placed on an SEM sample holder with N650 Planocarbon (Plano GmbH, Wetzlar, Germany). In biofilm test, three-dimensional (3D) images of the discs were merged using SEM pictures with different tilt angles (0°, 7°, and 15°) before and after removal of the degradation products.

### 2.7. Data Analysis

Statistical analyses were performed by one-way analysis of variance (ANOVA) in Origin 9.0G with the appropriate post hoc comparison test (Tukey's test). A *p* value <0.05 was considered significant. The graphs present the results as the mean value with the standard deviation (SD) as the error bars.

## 3. Results

### 3.1. Metallography and Microstructure

**Figure 2:**
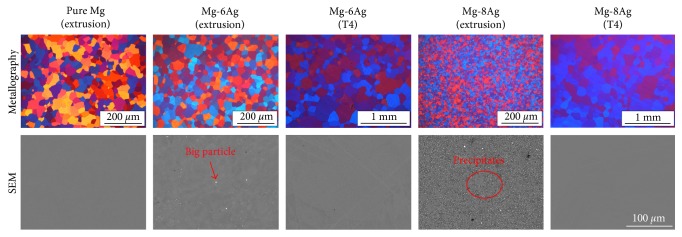
Metallography, distribution, and quantity of secondary phases and precipitates in the extruded Mg-Ag alloys before and after T4 treatment. The grain sizes of extruded pure Mg, extruded Mg-6Ag, T4-treated Mg-6Ag, extruded Mg-8Ag, and T4-treated Mg-8Ag are 29.9 ± 15.5, 28.2 ± 13.4, 154.5 ± 103.4, 7.9 ± 4.5, and 159.9 ± 89.7 *μ*m, respectively. The SEM in BSE (back-scattered electron) mode was used. The ratios of second phases and precipitates in the extruded Mg-6Ag and Mg-8Ag are 0.2% and 2.3%, respectively.

**Figure 3:**
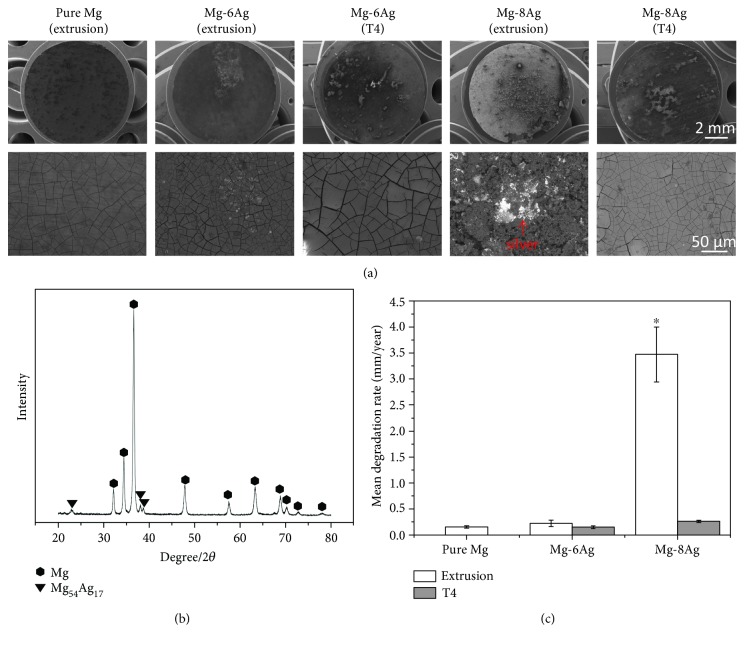
(a) Surface morphology of discs after the immersion test. The images of the local details are in BSE mode. (b) XRD pattern of the extruded Mg-8Ag alloy. (c) The mean degradation rate of pure Mg and Mg-Ag alloys in CCM in the cell culture conditions. The mean degradation rate of the extruded Mg-8Ag is significantly higher than the others. The “^∗^” indicates a significant difference compared with the other values, *p* < 0.05.

The actual elemental composition of the Mg-Ag alloys is listed (Table 1). Pure Mg has a similar grain size as extruded Mg-6Ag. Extruded Mg-8Ag has finer grains (average grain size (AGS) = 7.9 ± 4.5 *μ*m) than extruded Mg-6Ag (AGS = 28.2 ± 13.4 *μ*m). However, the grain size increased obviously after the T4 treatment and showed a nonuniform trend, but the microstructures of Mg-6Ag and Mg-8Ag became similar after T4 treatment (Figure 2). According to the XRD patterns, the precipitates in the extruded Mg-6Ag and Mg-8Ag are Mg_54_Ag_17_ (Figure 3(b)). Most of the precipitates are distributed along the grain boundaries of the extruded Mg-Ag alloys, as shown in the SEM images. There is a small amount of precipitate (0.2%) in the extruded Mg-6Ag and a large amount (2.3%) in the extruded Mg-8Ag. Some larger particles can be found in the extruded Mg-6Ag and Mg-8Ag that are residuals of the secondary phase after the homogenization treatment of the ingots. After T4 treatment, nearly all the secondary phases and precipitates in the extruded Mg-Ag alloys dissolved into the alloys.

### 3.2. Morphology and Degradation Rate

After the immersion test, the surface condition of the discs can be observed (Figure 3(a)). Some black or white dots, which are degradation products, are present on the surface of the pure Mg and Mg-Ag alloys in low-magnification images. However, the extruded Mg-8Ag disc showed more severe degradation than the other discs. Loose degradation products and accumulated silver are observed on the surface. There is also some accumulated silver on the surface of the extruded Mg-6Ag disc. However, there are only cracks on the pure Mg and T4-treated Mg-Ag discs after the degradation layer is dehydrated.

The addition of silver led to a slight increase in the degradation rate of Mg-6Ag alloy compared to pure Mg (Figure 3(c)). A further increase in silver significantly enhanced the degradation to 3.47 mm/year. This effect could be drastically reduced by T4 treatment, which led to degradation rates of less than 0.5 mm/year. In this case, the degradation rate increased linearly with the amount of silver.

### 3.3. Cytocompatibility

#### 3.3.1. MTT

The pH and osmolality of the extracts from the pure Mg and extruded Mg-Ag alloys were elevated compared to the CCM (Table 2). More Mg existed in the extracts than in CCM, but the concentration of Ca in the extracts decreased (Table 3). Magnesium/calcium phosphates formed in the degradation layer during degradation [52–55]. After T4 treatment, the osmolality of the Mg-Ag alloy extracts was lower than that of the pure Mg and extruded Mg-Ag extracts. The concentrations of Mg, Ca, and Ag in the primary extracts from extruded Mg-Ag were higher than those from T4-treated Mg-Ag.

**Figure 4:**
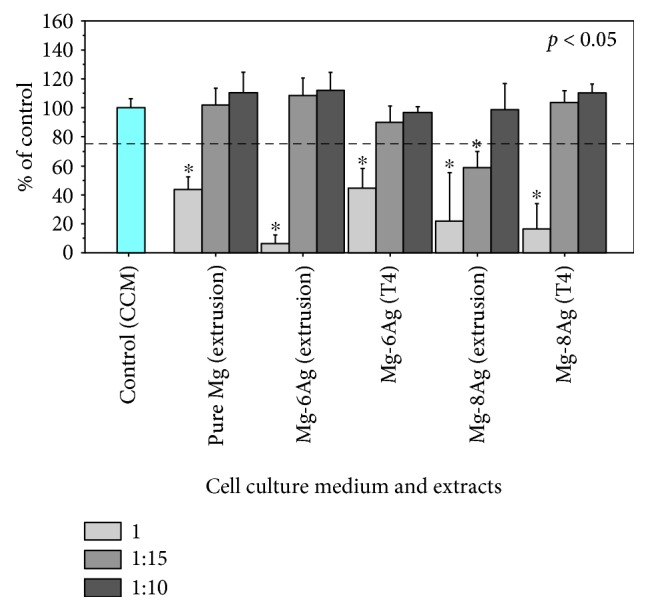
Viability of human primary osteoblasts determined by MTT assay in the primary, 1 : 5, and 1 : 10 extracts. The dotted line marks 75% cell viability, which indicates no potential cytotoxicity [58]. The “^∗^” indicates statistically significant difference at *p* < 0.05 versus the control group (CCM).

In the MTT assay, all primary extracts, including pure Mg extract, showed cytotoxicity compared with CCM because of high pH and osmolality (Figure 4). Their values were below the cytotoxic limit of 75% cell viability. After fivefold dilution, most of the extracts did not show cytotoxicity, except for the extract of the extruded Mg-8Ag, which also did not reach the level of 75% because the silver concentration was still higher than the tolerance of human primary osteoblasts. After fivefold and tenfold dilutions, the extract from T4-treated Mg-8Ag showed good cytocompatibility as did the diluted pure Mg extracts and CCM, although all primary extracts exhibited cytotoxicity to human primary osteoblasts.

#### 3.3.2. Live/Dead Staining and Adhesion Test

**Figure 5:**
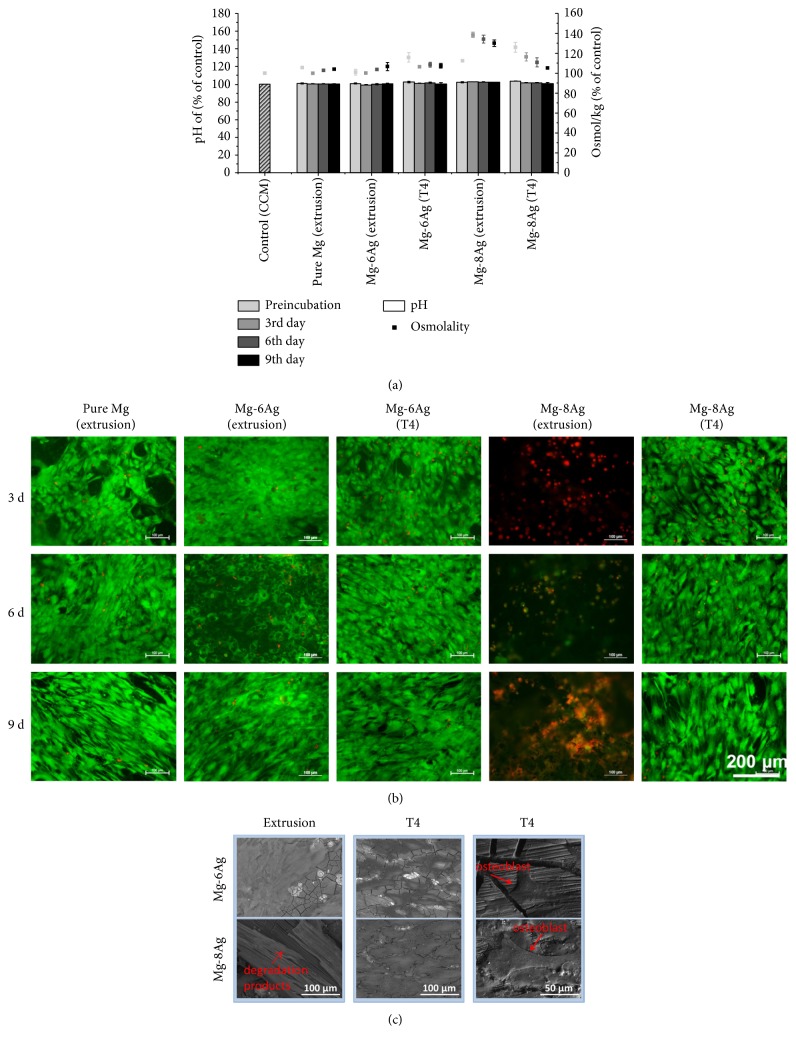
(a) Variation of pH and osmolality during the incubation. (b) Live/dead staining of human primary osteoblasts on pure Mg and Mg-Ag alloys. The clear green parts represent living osteoblasts and the red dots dead osteoblasts. (c) SEM images of the adhesion tests of human primary osteoblasts. The first and second vertical rows show thick cell layers covering the surface after 9 days. The third vertical row shows the status of a single osteoblast attached to the degradation layer after 3 days.

The pH and osmolality were measured after preincubation for 24 hours and culturing human primary osteoblasts on the samples for 3, 6, and 9 days. The pH of the pure Mg and T4-treated Mg-Ag discs decreased gradually over time (Figure 5(a)). The osmolality of the extract from pure Mg was stable, and the osmolality of the extract from extruded Mg-6Ag increased slightly over time. However, the osmolality of the extract from extruded Mg-8Ag remained at a high level after preincubation, and it had nearly the highest pH and osmolality at all time points. The extracts of the T4-treated Mg-Ag discs had higher osmolality after preincubation, but the osmolality decreased rapidly over time.

The regions with the same cell density were selected for comparison after live/dead staining (Figure 5(b)). The degradation rates of the extruded pure Mg and Mg-6Ag were much lower than those of the extruded Mg-8Ag in CCM in the cell culture conditions according to the pH and osmolality (Figure 5(a)). Human primary osteoblasts can survive and attach to extruded pure Mg, extruded Mg-6Ag, and T4-treated Mg-6Ag discs. After 3 days, on pure Mg and Mg-6Ag, some dead osteoblasts were detected. After 6 days, the number of dead osteoblasts decreased. After 9 days, no differences were detected between the viability of human primary osteoblasts on pure Mg and Mg-6Ag discs. However, the extract from the extruded Mg-8Ag always had the highest average pH and osmolality, which indicates a faster degradation rate. The pH and osmolality near the surface of the extruded Mg-8Ag discs were higher, so many bubbles formed on the surface and a large amount of silver was released. As a result, no human primary osteoblasts attached to and survived on the extruded Mg-8Ag discs. After T4 treatment, the pH and osmolality of Mg-8Ag discs were lower and showed better cytocompatibility than before. Human primary osteoblasts can attach and proliferate on T4-treated Mg-8Ag discs as well as on pure Mg and Mg-6Ag discs, but a slightly higher amount of dead cells was observed in the initial stage (after 3 days). Cell layers and the details of human primary osteoblasts could be observed on all surfaces of the Mg-Ag discs, except for the extruded Mg-8Ag disc, where there are only degradation products (Figure 5(c)).

### 3.4. Biofilm Test

#### 3.4.1. Viability of Bacteria

**Figure 6:**
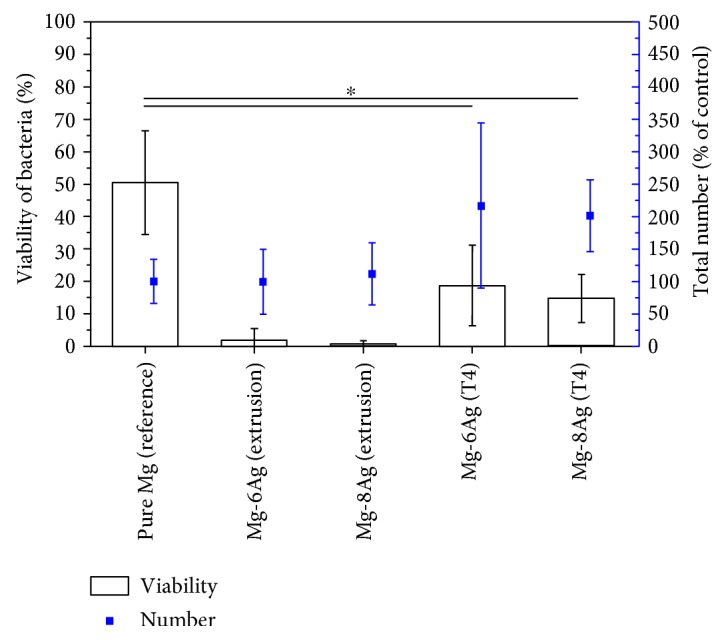
Viability of bacteria and total bacteria on the discs. From left to right—extruded pure Mg, extruded Mg-Ag alloys and T4-treated Mg-Ag alloys after extrusion. Pure Mg is the reference for the total number of bacteria. The “^∗^” means that pure Mg has a statistically significant difference in viability at *p* < 0.05 versus T4-treated Mg-Ag discs.

The extruded Mg-Ag alloys showed the best antibacterial effect (Figure 6). The viability of bacteria was much lower on the Mg-Ag alloys than on pure Mg. There was a relative high viability of bacteria on pure Mg of 50.35%. However, the viability on T4-treated Mg-6Ag and Mg-8Ag discs was 18.64% and 14.75%, respectively, which was significantly lower than that on pure Mg. In addition, more bacteria were observed on T4-treated Mg-Ag discs than on the extruded Mg-Ag discs.

#### 3.4.2. Biofilm Culture

**Figure 7:**
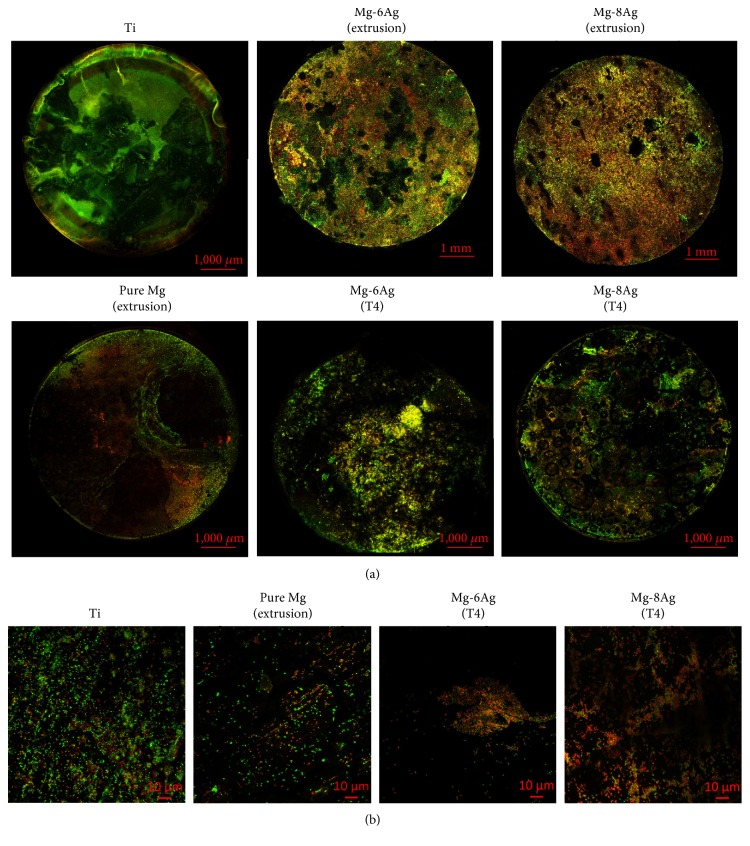
(a) Overview of biofilm formation. (b) Details of bacteria on discs.

In the biofilm test, incubation for 15 hours can form an initial biofilm on Ti discs, which were set as the negative control for internal evaluation. A nearly complete young biofilm can be observed on the Ti disc (Figure 7(a)). A large amount of live bacteria on Ti disc could be observed in the high-magnification images (Figure 7(b)). The total amount of bacteria on pure Mg is obviously lower than on Ti disc. Extruded Mg-Ag discs showed local pitting degradation and had a faster degradation rate in BCM with a constant pH (7.2) than in CCM (Figure 7(a)). Many dead bacteria were present on the extruded Mg-Ag discs based on the overview pictures. However, the overview of the biofilm showed no obvious difference between pure Mg and T4-treated Mg-Ag discs. By judging from the high-magnification images (Figure 7(b)), details of live and dead bacteria on the T4-treated Mg-Ag alloys were shown. Most of the bacteria on the surface of the T4-treated Mg-Ag discs were dead compared to the bacteria on pure Mg.

#### 3.4.3. Surface Morphology after the Biofilm Test in the Flow System

**Figure 8:**
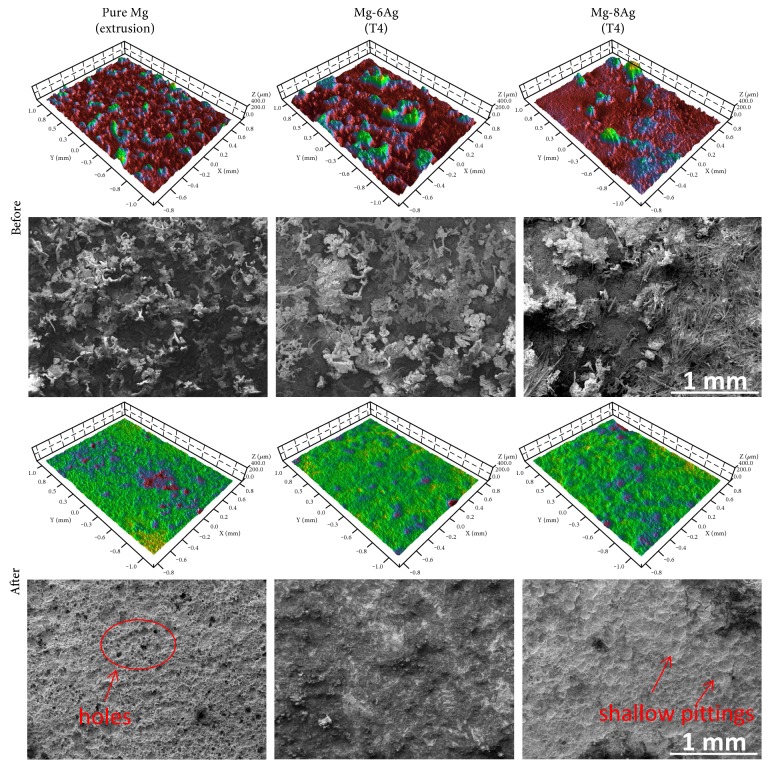
3D images and surface conditions (SEM) before and after removal of the degradation products. The average roughness (Sa) of pure Mg, Mg-6Ag, and Mg-8Ag are 8.68 ± 0.8, 6.42 ± 0.42, and 8.88 ± 1.92, respectively. The developed interfacial areas (Sdr) are 37.87 ± 1.44, 19.26 ± 2.72, and 22.39 ± 2.23.

The 3D images of pure Mg and T4-treated Mg-Ag discs before and after removal of the degradation products are shown (Figure 8). The surfaces of the discs with degradation products appear coarse. It can be observed that many degradation products are present on the surface of the pure Mg and Mg-Ag discs. There are also some needle-like crystals on the T4-treated Mg-6Ag disc and many needle-like crystals on the T4-treated Mg-8Ag disc, so they look very rough in the 3D images. However, after removal of degradation products, the peaks disappeared and many degradation pits were revealed, especially on pure Mg, where a porous surface was exposed. However, only shallow and broad pits were observed on the surface of T4-treated Mg-Ag discs, which indicates a more homogeneous degradation mechanism. The average roughness (Sa) of pure Mg, T4-treated Mg-6Ag, and T4-treated Mg-8Ag are 8.68 ± 0.8, 6.42 ± 0.42, and 8.88 ± 1.92, respectively, and the developed interfacial areas (Sdr) are 37.87 ± 1.44, 19.26 ± 2.72, and 22.39 ± 2.23, respectively. Therefore, the Sa of pure Mg and T4-treated Mg-Ag alloys is on the same level, and the Sdr of the T4-treated Mg-Ag alloys is significantly lower than that of the pure Mg at *p* < 0.05. Therefore, the T4-treated Mg-Ag alloys have less contact area with the medium than pure Mg during degradation.

## 4. Discussion

Silver release plays a key role in eliminating multiple bacteria and preventing biofilm formation [23]. The novel point is that the addition of silver to pure Mg significantly improved the antibacterial effect in a dynamic environment because silver was released from the matrix continuously during the whole degradation of Mg-Ag alloy as an orthopedic implant. In a previous study with cast material, the effect of silver addition could already be demonstrated [56]. However, cast magnesium alloys would never be used to produce implants. Therefore, in this study, extruded Mg-Ag alloys were evaluated. Extrusion generally led to a finer microstructure and was associated with lower degradation rates, imbalancing the silver release. Therefore, we evaluated higher silver contents in this study. We found that Mg-Ag alloys possessed much higher degradation rates when the silver content reached in magnesium 8 wt%. The high pH, osmolality, and silver concentration had negative effects on the viability of human primary osteoblasts, although good antibacterial properties were shown in dynamic conditions. T4 heat treatment decreased the degradation of Mg-Ag alloys. As a result, human primary osteoblasts could survive on the surface of T4-treated Mg-Ag alloys. Meanwhile, the Mg-Ag alloys still had a much better antibacterial effect than pure Mg.

Silver has a major influence on the degradation behavior of Mg-Ag alloys. Since it has low solubility in magnesium at ambient temperature [41], more secondary phases or precipitates exist in Mg-Ag alloys when the silver content is higher [56]. Both the amount and distribution of precipitates can affect the degradation behavior due to the principle of microgalvanic corrosion [12, 54]. The degradation rate increases linearly with increasing quantity of precipitates and the precipitates can cause localized degradation phenomenon [57]. For example, the extruded Mg-8Ag had higher degradation rate than the extruded Mg-6Ag. T4 treatment near the eutectic temperature can eliminate the secondary phases and precipitates. Since the Mg_54_Ag_17_ precipitates did not have the ability to restrict the migration of grain boundaries at high temperature, the grains were enlarged during T4 treatment. The elimination of precipitates weakened galvanic corrosion, so the degradation rate decreased [12]. For pure Mg and T4-treated Mg-Ag alloys, they had different grain sizes and both of them had no precipitates inside. T4-treated Mg-6Ag had nearly the same degradation rate compared to pure Mg. T4-treated Mg-6Ag had bigger grains than pure Mg, so grain size was not the key factor to influence the degradation rate. T4-treated Mg-6Ag and T4-treated Mg-8Ag had no significant difference of grain size. However, T4-treated Mg-8Ag had higher degradation rate than T4-treated Mg-6Ag to some extent. In this case, solid solution of silver in magnesium influenced the degradation rate when it reached 8 wt%. The pure Mg and T4-treated Mg-Ag alloys had lower degradation rates than the extruded Mg-Ag alloys in CCM. T4-treated Mg-Ag alloys had more homogeneous and flatter degradation surfaces and lower pitting degradation trends than pure Mg in the flow chamber according to the 3D images, even though the low pH (7.2) of BCM had an adverse effect on the stability of the degradation layer [57]. The morphology difference was related to the solution of silver and microstructural changes via T4 treatment. Hence, the existence of precipitates mainly influenced the degradation behavior followed by solution of silver rather than grain size.

The degradation rate of Mg-Ag alloys can influence the pH and osmolality of the medium and silver release, which are closely related to cytocompatibility [43]. A high degradation rate largely leads to increased pH, osmolality, hydrogen generation and silver release. However, the pH will not always increase linearly with the degradation rate because of a “saturation effect”. The increment of osmolality is because of Mg ion release instead of Ca and other ions. According to the MTT and adhesion test, high pH, osmolality, and silver contents can cause cytotoxicity to human primary osteoblasts, for example, extruded Mg-8Ag. It is also not easy for cells or bacteria to attach to the surface due to the hydrogen generation, although good antibacterial properties can be obtained. For pure Mg, the cytotoxicity of the primary extract is caused by the high pH and osmolality. Similarly, pure magnesium cannot rely on a high pH to achieve its antibacterial effect, regardless of cytotoxicity, because high osmolality or magnesium ion concentration causes osmotic shock in human cells [58]. Therefore, a high degradation rate is not required of magnesium alloys used as orthopedic implants, and the degradation rate should be controlled to meet the cytotoxicity criteria first.

The biofilm assay was conducted under flowing conditions. The in vitro design of the dynamic system with large numbers of bacteria in the flowing medium represents harsh conditions, although the in vivo conditions normally show clearly lower bacteria concentrations. The flowing conditions and pH control system can exclude the pH effect of the corroding Mg alloys as much as possible. Pure Mg did not show satisfactory antibacterial properties under these conditions. Based on the viability of bacteria on pure Mg discs, there are still considerable numbers of live bacteria that should not be neglected. From the overview of the biofilm and bacteria distribution on the discs, it appears that pure Mg has the potential to form many colonies or a biofilm layer under suitable conditions, although the total amount of bacteria is less than that on the negative control groups [59]. Admittedly, the pH plays an important antibacterial role due to the alkaline environment created during degradation [9–11]. The alkaline environment has adverse effects on bacteria in static conditions. It is unclear whether pure Mg has sufficient antibacterial properties in these conditions in a dynamic environment, such as the human body.

There was a large amount of silver released from the extruded Mg-Ag alloys because of high degradation rate. As a result, they showed good antibacterial properties. However, the viability of bacteria was not as low as expected, for example, 99.9%. One reason is that a large portion of silver flowed away with the medium and another reason is that a large amount of bacteria (10^6^/mL) exists in the medium, which indicates harsh conditions. The T4-treated Mg-Ag alloys got better antibacterial properties than pure Mg. T4-treated Mg-8Ag released more silver (twofold) than T4-treated Mg-6Ag, but bacteria viability decreased only by 4 percent due to the flow system of the bioreactor. In this case, less-released silver ions reacted with the bacteria attached to the surface of the discs. According to the decreasing trend of bacteria viability, if we continue to increase the silver content in pure Mg, for example, 15 wt%, more effective antibacterial properties of Mg-Ag alloy could theoretically be achieved in the bioreactor. However, the degradation rate approaches its limit at an acceptable range for orthopedic implants [60].

Silver ions can bind strongly and build complexes with thiols, metallothionein, albumins, and macroglobulins in vivo [29, 61, 62]. The antibacterial properties of Mg-Ag alloys are related to the silver concentration in the infection site, which is determined by the amount of silver in the Mg-Ag alloys and its release rate. If only a small amount of silver was released, the remaining silver was not sufficient to inhibit bacteria. In contrast, if the amount of active silver ions released was greater, the antibacterial properties would be more effective [25], so it is better to alloy as much silver as possible in pure Mg to ensure effective antibacterial properties on the basis of a controllable degradation rate. However, the total silver in the Mg-Ag alloy should not exceed the amount that can cause argyria in the human body.

## 5. Conclusion

In this study, the relationship between the microstructure, silver content, degradation behavior, cytotoxicity, and antibacterial properties of Mg-Ag alloys was revealed. The microstructure has a strong influence on the degradation behavior of Mg-Ag alloys. The degradation rate will be high if there are many precipitates in the Mg-Ag alloys. To reach a lower degradation rate, the microstructure was adjusted via solution treatment (T4). As a result, precipitates dissolved into the magnesium matrix and the grains enlarged. T4-treated Mg-Ag alloys showed low degradation rate as pure Mg and more homogeneous degradation than pure Mg. The T4-treated Mg-Ag alloys had no discernible in vitro cytotoxicity to human primary osteoblasts compared with pure Mg. Moreover, the antibacterial properties depend on silver release. By increasing the silver content and controlling the degradation rate, the T4-treated Mg-Ag alloys showed good antibacterial properties in the bioreactor system with flow conditions and abundant bacteria inside.

## Figures and Tables

**Table 1:** Major and trace element composition of the Mg-Ag alloys.

Alloys	Ag wt%	Fe wt%	Cu wt%	Ni wt%	Mg wt%
Mg-6Ag	6.26	0.00205	0.00100	0.00108	Balance
Mg-8Ag	8.51	0.00184	0.00104	0.00106	Balance

**Table 2:** Increments of pH and osmolality of pure Mg and Mg-Ag alloys compared to those of CCM.

Extracts	pH	Osmol/kg
Pure Mg (extrusion)	0.815	0.107
Mg-6Ag (extrusion)	0.920	0.110
Mg-6Ag (T4)	0.955	0.066
Mg-8Ag (extrusion)	0.895	0.104
Mg-8Ag (T4)	0.965	0.068

**Table 3:** Calculated concentrations of elements in the extracts of extruded pure Mg and Mg-Ag alloys.

Extracts	Dilution	Concentration (mg/L)		
		Mg	Ca	Ag
Pure Mg (extrusion)	1	1210	27	<0.1
	1/5	258	65.4	<0.1
	1/10	139	70.2	<0.1
Mg-6Ag (extrusion)	1	1280	26	1.2
	1/5	272	65.2	0.24
	1/10	146	70.1	0.12
Mg-6Ag (T4)	1	1010	17	0.31
	1/5	218.8	62.6	0.062
	1/10	119.9	68.3	0.031
Mg-8Ag (extrusion)	1	1150	25	104
	1/5	246	65	20.8
	1/10	133	70	10.4
Mg-8Ag (T4)	1	930	15	0.64
	1/5	202.8	62.2	0.128
	1/10	111.9	68.1	0.064
Cell culture medium (CCM)		20	75	<0.1
